# Transcriptome Analysis of Endogenous Hormone Response Mechanism in Roots of *Styrax tonkinensis* Under Waterlogging

**DOI:** 10.3389/fpls.2022.896850

**Published:** 2022-06-06

**Authors:** Hong Chen, Qikui Wu, Ming Ni, Chen Chen, Chao Han, Fangyuan Yu

**Affiliations:** ^1^Collaborative Innovation Centre of Sustainable Forestry in Southern China, College of Forest Science, Nanjing Forestry University (NJFU), Nanjing, China; ^2^State Forestry and Grassland Administration Key Laboratory of Silviculture in Downstream Areas of the Yellow River, College of Forestry, Shandong Agricultural University, Tai’an, China

**Keywords:** waterlogging, phytohormones, differently expression genes, intolerance, crosstalk

## Abstract

As a promising oil species, *Styrax tonkinensis* has great potential as a biofuel due to an excellent fatty acid composition. However, frequent flooding caused by global warming and the low tolerance of the species to waterlogging largely halted its expansion in waterlogged areas. To explore endogenous hormones and phytohormone-related molecular response mechanism of *S. tonkinensis* under waterlogging, we determined 1-aminocyclopropane-1-carboxylic acid (ACC) and three phytohormone content (ABA, abscisic acid; SA, salicylic acid; IAA, indole-3-acetic acid) and analyzed the transcriptome of its seedlings under waterlogged condition of 3–5 cm. The sample collecting time was 0, 9, 24, and 72 h, respectively. It was concluded that ACC presented an upward trend, but other plant hormones showed a downward trend from 0 to 72 h under waterlogging stress. A total of 84,601 unigenes were assembled with a total length of 81,389,823 bp through transcriptome analysis. The GO enrichment analysis of total differentially expressed genes (DEGs) revealed that 4,637 DEGs, 8,238 DEGs, and 7,146 DEGs were assigned into three main GO functional categories in 9 vs. 0 h, 24 vs. 0 h, and 72 vs. 0 h, respectively. We also discovered several DEGs involved in phytohormone synthesis pathway and plant hormone signaling pathway. It was concluded that the decreased transcription of PYL resulted in the weak ABA signal transduction pathway. Moreover, decreased SA content caused by the low-expressed PAL might impact the resistance of *S. tonkinensis* seedlings under waterlogging stress. Our research may provide a scientific basis for the understanding of the endogenous hormone response mechanism of *S. tonkinensis* to waterlogging and lay a foundation for further exploration of the waterlogging defect resistance genes of *S. tonkinensis* and improving its resistance to waterlogging stress.

## Introduction

Global warming leads to the variation in precipitation patterns, inevitably resulting in extreme weather events, such as frequent flooding (Arnell and Gosling, 2014; Striker and Colmer, 2017). Flooding causes waterlogging (root system inundation) or full submergence (root and aerial system flooding) disasters on over 17 million km^2^ of land each year worldwide (Bailey-Serres and Voesenek, 2008; Voesenek and Sasidharan, 2013; Van Dongen and Francesco, 2015). Commonly, it is hard to predict waterlogging, due to the uncertainty of flooding in a transitory period (Ren et al., 2016). Waterlogging stress was defined as soil saturated with water and restricted from the air for a period, which induces hypoxic or anoxic soil conditions (Arif et al., 2021). It is estimated that the O_2_ diffusion rate in the atmosphere is over 10^4^ times than that in the waterlogged soil, heavily impairing gas exchange between plants and the atmosphere (Kaur et al., 2021). O_2_ deprivation caused by waterlogging stress shifts the energy mechanism from aerobic respiration to anaerobic fermentation, leading to energy dissipation (Finch-Savage et al., 2005; Bailey-Serres and Voesenek, 2008). Yu et al. (2015) reported that enzymes related to glycolysis and fermentation pathways would be stimulated by waterlogging. The exhaustion of O_2_ triggers reactive oxygen species (ROS) overproduced by roots (Das and Roychoudhury, 2014; Hossain et al., 2015). The excessive ROS accumulation attacks membrane lipids, which generates oxidative damage to proteins, nucleic acids, and lipids (Yin et al., 2009; Yang et al., 2011, 2015; Gao et al., 2015). In addition, the root system induces leaf stomatal closure under hypoxic conditions. Both enhanced resistance to diffusion of carbon dioxide (CO_2_) and declined chlorophyll content lead to the low efficiency of light harvesting, thereby reducing plant photosynthesis and seriously impacting plant production (Linkemer et al., 1998; Jacobsen et al., 2007; Yordanova and Popova, 2007; Ashraf, 2012; Amri et al., 2014).

To mitigate the impacts of adversities on plants under waterlogging pressure, plants carry out escape, quiescent, and self-regulating compensation strategies (Zhang et al., 2020). Previously, numerous excellent studies demonstrated that plants could develop adventitious roots for aerobic respiration and energy production (Jackson, 1985; Gibberd et al., 2001; Visser and Voesenek, 2005), generate metabolic energy by the glycolysis and ethanol fermentation pathways (Capon et al., 2009; Ismail et al., 2009; James et al., 2016), and trigger reactive oxygen scavengers to mitigate detrimental effects of ROS under waterlogging stress (Komatsu et al., 2013). In the response of plants, phytohormones also work as endogenous signaling elicitors in higher plants to bridge these response strategies against waterlogging stimuli (Kazan, 2015; Zhao Y. et al., 2018; Zaid et al., 2021). Costa et al. (2020) reported that auxins were essential for the formation of adventitious roots in *Arabidopsis thaliana* to combat flooding. Ethylene (ET) acts as a key regulator of the aerenchyma formation in rice (Fukao and Bailey-Serres, 2008). The crosstalk between the jasmonates and ET production also plays a vital role in forming and developing root and aerenchyma in response to waterlogging (Farhangi-Abriz and Ghassemi-Golezani, 2019). Phytohormones are molecules synthesized in plants at a low concentration and transported to demanding sites specifically, acting as chemical messengers under optimal or stressful circumstances. They play an essential role in cellular communication to regulate physiological and molecular processes, which are vital for the survival of plants under external and internal stresses (Went and Thimann, 1937; Vob et al., 2014; Kazan, 2015). Consequently, further exploration of the phytohormone regulatory mechanisms on waterlogging response is essential for plant survival and production.

*Styrax tonkinensis* (Pierre) Craib ex Hartwich belongs to Styracaceae family and Styrax genus. It is native to Laos, Vietnam, Cambodia, Thailand, and southern China (Pinyopusarerk, 1994). This species is a deciduous tree with high timber, seed oil, medicine, and ornamental value (Hieu et al., 2011; Xu and Yu, 2015; Burger et al., 2016). It also has great potential as a biofuel due to its excellent fatty acid composition and over 50% oil content in its kernels (Wu et al., 2020). Benzoin, a colorless or white crystal, is a commercial product from the resin of *S. tonkinensis*, which displays as an alleviator for treating leukemia in modern pharmaceuticals (Wang et al., 2006). To date, previous studies regarding *S. tonkinensis* have mainly focused on woody biodiesel, oil accumulation, and floral scent (Xu and Yu, 2015; Wu et al., 2020). According to the pre-experiment (16 waterlogged *S. tonkinensis* seedlings), it was discovered that *S. tonkinensis* seedlings are susceptible to waterflooding since their leaves began to wilt and drop on the third day of waterlogging. Moreover, the mortality of these seedlings reached 100% by the 5th day of waterlogging. However, less is known about the response of phytohormones and the molecular regulatory mechanism of this species to waterlogging. Zhou et al. (2021) discovered that the content of abscisic acid (ABA) and gibberellin (GA) changed in soybean seeds under waterlogging. Besides, Khan et al. (2020) integrated the functions of ET and its crosstalk with other phytohormones in plants in response to flooding. Similarly, it was hypothesized that the collaboration of these phytohormones played a significant role in flooding stress tolerance in *S. tonkinensis*. In this study, we also explored the changes in phytohormone content and phytohormone-related gene expression in *S. tonkinensis* under waterlogging stress at four time points *via* both physiological and molecular approaches. The study aimed to: (1) determine the dynamic pattern of phytohormones under waterlogging; (2) assess transcriptional profiles corresponding to phytohormone biosynthesis and phytohormone signal transduction; and (3) discuss the interaction of these phytohormones. Eventually, the purpose of our study was to enhance the understanding of these regulatory mechanisms, which are imperative for facilitating the survival rate of *S. tonkinensis* in flooding seasons.

## Materials and Methods

### Plant Material and Waterlogging Treatment

The *S. tonkinensis* seeds are derived from Pingxiang, Jiangxi Province, China, and plug seedlings were cultivated in a greenhouse at the Xiashu Forest Farm, Zhenjiang, Jiangsu Province, China (118°40′E, 31°41′N). The young plug seedlings with an average height of 15 cm were transplanted in permeable non-woven containers containing a medium of organic matter: perlite: vermiculite (v/v: 6:2:2) to continuous growth through common water and nutrient management. The waterlogging treatments were started at 8 a.m. on 5 September 2021 when seedlings reached the average height of 55 cm. The seedlings were planted plot-in-plot (Supplementary Figure 1A). The inside basin is the permeable non-woven container, and the outside basin is a water-proof lotus plot. During the experimental period, the daily average temperature varied between 25.9 and 29.6°C. Relative humidity (RH) ranged from 62 to 76%. The daylight varied between 12.6 and 12.7 h with 13,316 KJ/m^2^ average daily solar irradiation. The waterlogging treatment lasted for 4 days, and the water surface elevated the matrix by 3–5 cm. A total of 16 seedlings were destructively collected for each sample counting the survival rate every day. The primary root samples (Supplementary Figure 1B) were collected at 0, 9, 24, and 72 h after waterlogging treatment, immediately frozen in liquid nitrogen and kept at –80°C refrigerators for further processing. The sample without waterlogging treatment (0 h) was set as the control group. A number of three biological replicates for each analytical experiment of the transcriptome analyses and the quantitative real-time PCR (qRT-PCR) experiments were harvested.

### Endogenous Phytohormone Extraction and Determination

The content of three phytohormones including salicylic acid (SA), abscisic acid (ABA), and indole-3-acetic acid (IAA) was determined. The endogenous phytohormones were extracted using acetonitrile solution. About 0.5 g of fresh root samples was first mixed with acetonitrile solution at a volume ratio of 1:10, followed by centrifugation, purification, and nitrogen blowing. The measurements were taken using the AGILENT 1,290 high-performance liquid chromatography (HPLC) (AGILENT Company, Palo Alto, CA, United States) series SCIEX-6500Qtrap mass spectrometer (MS/MS) (AB Company, Framingham, MA, United States). Chromatographic separation of five types of phytohormones was performed using a Poroshell 120 SB-C18 reverse-phase chromatography column (2.1 mm × 150 mm, 2.7 μm). Phase A (methanol/0.1% formic acid solution) and phase B (water/0.1% formic acid solution) of HPLC-MS/MS were utilized to conduct gradient elution at a constant flow rate of 0.3 ml/min at 30°C. In this experiment, multiple reaction detection (MRM) scan mode was applied to detect the substance. For the substances to be tested, 2 or more fragment ions were determined. The ratio of their peak time and response value corresponded to the standard substance was analyzed, to identify the detected objects. The standard substances that include D-ABA, D-IAA, and D-SA were purchased from Sigma-Aldrich (Shanghai, China) to draw the standard curve to qualify the detected objects.

### 1-Aminocyclopropane-1-Carboxylic Acid Extraction and Determination

Approximately 0.2 g of fresh root samples was collected to determine 1-aminocyclopropane-1-carboxylic acid (ACC). Under hypoxia conditions, plants can generate the ET precursor (ACC) in roots and vertically transfer it to the xylem. Subsequently, ACC would be oxidized to ET in shoots. ACC would be determined to replace ET due to that ET is volatile and hard to be detected (Banga et al., 2010; Bradford et al., 2018). It was isolated by 4°C precooling deionized water for 2 h. After centrifugation and purification, 2 μl of samples was displaced into AGILENT 1,290 high-performance liquid chromatography series 6420A mass spectrometer (AGILENT Company, Palo Alto, CA, United States) for further detection. Poroshell 120 SB-C18 reverse-phase chromatography column (2.1 mm × 150 mm, 2.7 μm) was also applied. Phase A (acetonitrile solution) and phase B (water/0.1% formic acid solution) of HPLC-MS/MS were utilized to conduct gradient elution at 35°C. The ACC standard was bought from J&K Scientific Ltd. (Beijing, China).

### RNA Extraction and cDNA Library Construction

Root samples collected at 0, 9, 24, and 72 h were used for transcriptome analyses. Total RNAs were extracted using the Plant RNA Kit (Ambion) following the manufacturer’s protocol. RNA integrity was evaluated using the Agilent 2100 Bioanalyzer (Agilent Technologies, Santa Clara, CA, United States). The samples with RNA integrity number (RIN) ≥ 7 were subjected to the subsequent analysis. The libraries were constructed using TruSeq Stranded mRNA LT Sample Prep Kit (Illumina, San Diego, CA, United States) according to the manufacturer’s instructions. Then, these libraries were sequenced on the Illumina Hiseq 4000 Sequencing platform, and 125/150-bp paired-end reads were generated.

### Quality Control and *de novo* Assembly

The transcriptome sequencing and analysis were conducted by OE Biotech Co., Ltd. (Shanghai, China) using Illumina Hiseq 4000 Sequencing platform. Raw data (raw reads) were processed using Trimmomatic (Bolger et al., 2014). The reads containing poly-N and the low-quality reads were removed to obtain the clean reads. After removing adaptor and low-quality sequences, the clean reads were assembled into expressed sequence tag clusters (contigs) and *de novo* assembled into the transcript using Trinity (Grabherr et al., 2013) in the paired-end method. The longest transcript was chosen as a unigene based on the similarity and length of a sequence for subsequent analysis.

### Functional Annotation

The function of the unigenes was annotated by alignment of the unigenes with the NCBI non-redundant (NR), SwissProt, and clusters of orthologous groups for eukaryotic complete genome (KOG) databases using BLASTx (Altschul et al., 1990) with a threshold E-value of 10^–5^. The proteins with the highest hits to the unigenes were used to assign functional annotations thereto. Based on the SwissProt annotation, Gene Ontology (GO) classification was performed by the mapping relation between SwissProt and GO term. The unigenes were mapped to the Kyoto Encyclopedia of Genes and Genomes (KEGG) (Kanehisa et al., 2008) database to annotate their potential metabolic pathways.

### Differential Expression Analysis of Unigenes

The DESeq2 method was applied to standardize the count number of each sample gene, and the base mean value was used to estimate the expression level, which was calculated as fragments per kilobase per million mapped reads (FPKM). Besides, the multiple of difference was calculated, and negative binomial (NB) distribution test was used to test the significance of the difference. Finally, the differential protein-coding genes were screened according to the different multiple and different significance test results (Love et al., 2014). Expression levels of unigenes were calculated. The DEGs between different time points were identified with *p* < 0.05 and | log2FC| > 1 (Anders and Huber, 2010).

### Quantitative Real-Time PCR Analysis

To validate the RNA-seq results, a total of eight transcripts ([IAA], PYL, EBF1_2, EIN3, MYC2, NPR1, AHP, and AUX1/LAX), which were associated with phytohormones biosynthesis at each of sampling points, were verified by qRT-PCR. The primers for each DEG are displayed in Table 1. All reactions were done on a StepOne Real-Time PCR System using SYBR Green Dye (Applied Biosystems, Foster City, CA, United States; Takara, Dalian, China). Relative gene expression was evaluated using the 2^–ΔΔ^*^Ct^* method with 18S ribosomal RNA as an internal control.

**TABLE 1:** Primers used for quantitative real-time PCR (qRT-PCR) analysis.

Gene id	Gene name	Forward primer (5′–3′)	Reverse primer (3′–5′)
TRINITY_DN25603_c0_g1_i3_1	IAA	TACCTGAGGAAGGTTGATCT	TGCAAGAGTCTGTGAACATT
TRINITY_DN18102_c0_g1_i1_2	PYL	AATCTCCAATCTCTCGCTCA	TCGTAATATCATCGGAACTGG
TRINITY_DN28542_c0_g1_i1_1	EBF1_2	AACTGTCCCAATCTGACATC	GCCATAATGTCCAACAACAG
TRINITY_DN31950_c0_g1_i1_1	EIN3	AAGTTAGCAAGCCAGTCTAC	GAAGCTGAATTACGGGAGAT
TRINITY_DN34422_c0_g1_i1_4	MYC2	GTTTTACAACTGCGAGAGAG	TCTAAACCGACCATTATGCC
TRINITY_DN31317_c0_g1_i5_1	NPR1	TTGTGACCCCTCAATTTACC	AAGAAACCCACCACATAGAC
TRINITY_DN30151_c0_g1_i1_4	AHP	CACCAACAGACTGTGGATTA	CCATCCTGAACAGAGTTTCA
TRINITY_DN43795_c0_g3_i2_4	AUX1/LAX	CATCAGCGTTCTGTACATTG	GCTCCGAATATGTATGTCCA

### Statistical Analysis

The data analysis included a basic descriptive analysis followed by an analysis of variance (ANOVA). Duncan and Pearson R correlation tests were performed using SPSS 23.0 for Windows (SPSS Science, Chicago, IL, United States). The *p*-values less than 0.05 and 0.01 were both considered to indicate significance between groups. For the elaboration of graphs, Origin 2018 (OriginLab, Northampton, MA, United States) was used.

## Results

### The Analyses of 1-Aminocyclopropane-1-Carboxylic Acid and Endogenous Phytohormones Contents

**FIGURE 1:**
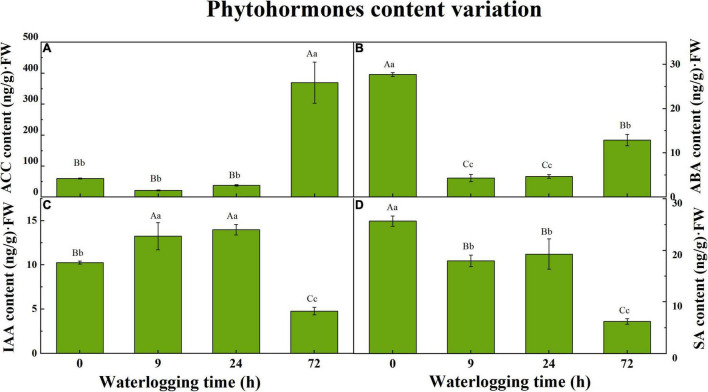
The variation in 1-aminocyclopropane-1-carboxylic acid (ACC) content **(A)**, abscisic acid (ABA) content **(B)**, indole-3-acetic acid (IAA) content **(C)**, and salicylic acid (SA) content **(D)** in roots of *Styrax tonkinensis* seedlings under waterlogging stress after 0, 9, 24, and 72 h, respectively. Different lowercase letters within each graph indicate significant differences (*p* < 0.05) among the samplings, whereas different capital letters within each graph indicate significant differences (*p* < 0.01) among the samplings. FW, fresh weight.

1-Aminocyclopropane-1-carboxylic acid and three phytohormones at 0, 9, 24, and 72 h were quantified by the HPLC-MS/MS method. The group of 0 h was regarded as the control group to compare with waterlogged groups. For each time point, it had three biological replicates. ACC, a precursor of ET, was sharply raised to 369.2 ± 38.35 Aa ng/g⋅FW after 72 h of waterlogging stress. During the first day of waterlogging, a slight variation in ACC content in roots appeared among three sampling points (Figure 1A). Compared to the control group (0 h), the ABA content in roots under waterlogging treatment decreased, especially for the seedlings that experienced 9 and 24 h of waterlogging. Nonetheless, ABA content was pointing to a bit of a rebound on the third day (12.88 ± 0.75 Bb ng/g⋅FW), which had significant differences from other groups (Figure 1B). The IAA content was elevated substantially after 9 h first and dropped subsequently on the third day (Figure 1C). Waterlogging inhibited SA accumulation in *S. tonkinensis* roots. Compared to the seedlings without waterlogging, SA content descended by approximately 30% during the first day of waterlogging and rapidly dropped by 76% with the aggravation of flooding stress on the third day (Figure 1D).

### Quality Control, *de novo* Assembly, and Total Gene Expression

**FIGURE 2:**
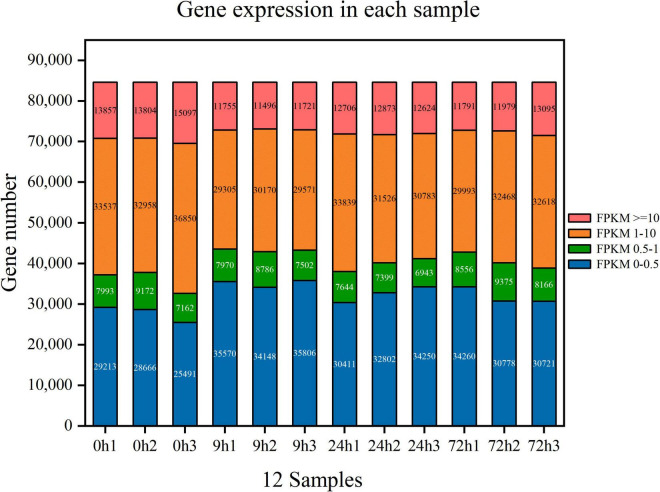
Gene expression in each sample of *Styrax tonkinensis* seedlings under waterlogging stress after 0, 9, 24, and 72 h, respectively. Different colors in the figure represent different ranges of FPKM values. The horizontal axis is each sample, and the vertical axis is the number of protein-coding genes.

A total of 78.73 G of clean data were obtained after transcriptome sequencing of 12 samples was completed. The effective data amount of each sample ranged from 6.12 to 6.92 G, Q30 bases ranged from 94.65 to 95.51%, and the average GC content was 46.12% (Table 2). A total of 84,601 unigenes were spliced with a total length of 81,389,823 bp and an average length of 962.04 bp (Table 3). Concerning the mapping statistics, the total reads of each sample ranged from 43,206,426 to 48,601,426 with a ratio of 85.53–89.67% when comparing reads to unigenes (Table 4). The 12 samples from 0, 9, 24, and 72 h were analyzed through the DESeq2 method to determine FPKM (Figure 2). For the 0-h group, the gene number of FPKM ranged from 1 to 10 was 33,537, 32,958, and 36,850 for each sample, respectively. 0h3 sample differenced with 0h1 and 0h2 samples. In the 9-h group, the gene number of FPKM over 10 and FPKM ranged from 1 to 10 was also similar between each sample.

**TABLE 2:** Statistic of raw data and clean data.

Sample	Raw reads	Raw bases	Clean reads	Clean bases	Valid bases (%)	Q30 (%)	GC (%)
0h1	47.18M	7.08G	46.58M	6.62G	93.48	95.35	46.07
0h2	45.60M	6.84G	45.01M	6.42G	93.85	95.39	46.25
0h3	49.32M	7.40G	48.60M	6.92G	93.51	95.27	46.57
9h1	46.73M	7.01G	46.13M	6.57G	93.75	95.51	46.54
9h2	44.33M	6.65G	43.21M	6.12G	91.96	94.79	46.00
9h3	47.14M	7.07G	45.78M	6.46G	91.38	94.65	45.75
24h1	47.16M	7.07G	46.09M	6.53G	92.33	94.88	46.19
24h2	48.04M	7.21G	47.32M	6.73G	93.33	95.09	46.62
24h3	48.74M	7.31G	48.03M	6.83G	93.46	95.12	46.48
72h1	44.44M	6.67G	43.59M	6.17G	92.62	94.88	45.94
72h2	48.28M	7.24G	47.49M	6.76G	93.40	94.94	45.55
72h3	47.63M	7.15G	46.52M	6.60G	92.32	94.87	45.54

**TABLE 3:** Transcriptome assembly statistics.

Term	All	≥500 bp	≥1,000 bp	N50	Total length	Max length	Min length	Average length
Unigene	84,601	47,770	26,066	1,507	81,389,823	11,108	301	962.04

**TABLE 4:** Mapping statistics of each sample.

Sample	Total reads	Total mapped reads	Multiple mapped	Uniquely mapped	Reads mapped in proper pairs
0h1	46576668 (100.00%)	40390325 (86.72%)	12023736 (25.81%)	28366589 (60.90%)	37672110 (80.88%)
0h2	45008924 (100.00%)	39308111 (87.33%)	11186912 (24.85%)	28121199 (62.48%)	36813420 (81.79%)
0h3	48601426 (100.00%)	41599984 (85.59%)	11062895 (22.76%)	30537089 (62.83%)	38995002 (80.23%)
9h1	46128342 (100.00%)	40932792 (88.74%)	13658621 (29.61%)	27274171 (59.13%)	38184968 (82.78%)
9h2	43206426 (100.00%)	38086045 (88.15%)	12824137 (29.68%)	25261908 (58.47%)	35496800 (82.16%)
9h3	45777808 (100.00%)	40509829 (88.49%)	13111183 (28.64%)	27398646 (59.85%)	37840932 (82.66%)
24h1	46093996 (100.00%)	41332633 (89.67%)	13734400 (29.80%)	27598233 (59.87%)	38580490 (83.70%)
24h2	47320162 (100.00%)	42197354 (89.17%)	13952885 (29.49%)	28244469 (59.69%)	39361940 (83.18%)
24h3	48026740 (100.00%)	42736667 (88.99%)	14102307 (29.36%)	28634360 (59.62%)	39851508 (82.98%)
72h1	43588700 (100.00%)	37280136 (85.53%)	12415326 (28.48%)	24864810 (57.04%)	34804146 (79.85%)
72h2	47488924 (100.00%)	41279867 (86.93%)	13424842 (28.27%)	27855025 (58.66%)	38504860 (81.08%)
72h3	46516460 (100.00%)	39978725 (85.95%)	13103239 (28.17%)	26875486 (57.78%)	37292532 (80.17%)

The quality control was applied after data output statistics *via* box-whisker plot and the purpose of principal component analysis (PCA). In the transcriptome results of *S. tonkinensis* seedlings under waterlogging stress, phytohormone-related unigenes were selected to conduct further analysis. The related unigenes were renewedly managed to draw Supplementary Figure 2. It was indicated the distribution of gene expression levels in Supplementary Figure 2A. The median gene expression amount for each group and the height of these boxes were basically at the same level. Even though these samples originated from the same tissue (primary roots) of *S. tonkinensis* seedlings, three biological replicates in each group still had a slight gene expression difference. PCA is to assess the biological repeatability of samples within groups and differences between groups. As shown in Supplementary Figure 2B, the long distance in the graph between each group illustrated obvious group differences. It was remarkable that the 24h1 sample was close to the 72-h group, manifesting that the 24h1 sample was more susceptible to waterlogging stress. For each group, the biological repeatability performed decently as the distance in the graph between each sample in a group was close.

### The Gene Ontology Enrichment Analysis of Total Differentially Expressed Genes and Phytohormone-Related Differentially Expressed Genes Between Waterlogged Groups and the Control Group

**FIGURE 3:**
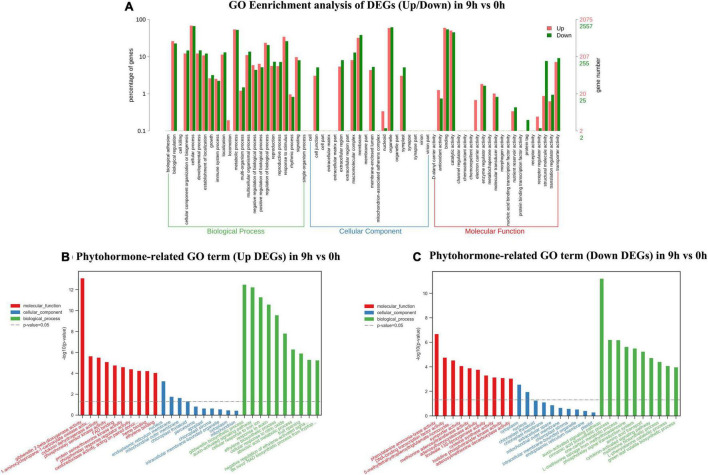
Gene ontology (GO) enrichment analysis of up/down differentially expressed genes (DEGs) **(A)**, phytohormone-related GO terms (up DEGs) **(B)**, and phytohormone-related GO terms (down DEGs) **(C)** in 9 vs. 0 h.

In this section, total DEGs and DEGs related to plant hormones in 9 vs. 0 h, 24 vs. 0 h, and 72 vs. 0 h were analyzed *via* GO classification, respectively (Supplementary Table 2). Massive GO pathways were filtered to the top 30 of enrichment for the following analysis. In the 9 vs. 0-h comparison, a total of 4,637 DEGs were assigned to three main GO functional categories, including biological process (3,752, 80.91%), cellular component (4,046, 87.25%), and molecular function (4,032, 86.95%). The biological process, cellular component, and molecular function were independently assigned into 10 sub-categories with the majority of unigenes in “cellular process” (up 68.24%, down 66.21%), “organelle” (up 58.84%, down 61.75%), and “binding” (up 59.52%, down 54.20%). As shown in Figures 3B,C and a total of 167 DEGs related to plant hormones were separately annotated to biological process (155, 92.81%), cellular component (125, 74.85%), and molecular function (143, 85.63%). In the biological process, the upregulated unigenes in “auxin-activated signaling pathway” and “ethylene biosynthetic process” ranked 2nd and 5th with 14 and 6 unigenes, respectively. A total of 12 downregulated unigenes in “auxin-activated signaling pathway” ranked top 1.

**FIGURE 4:**
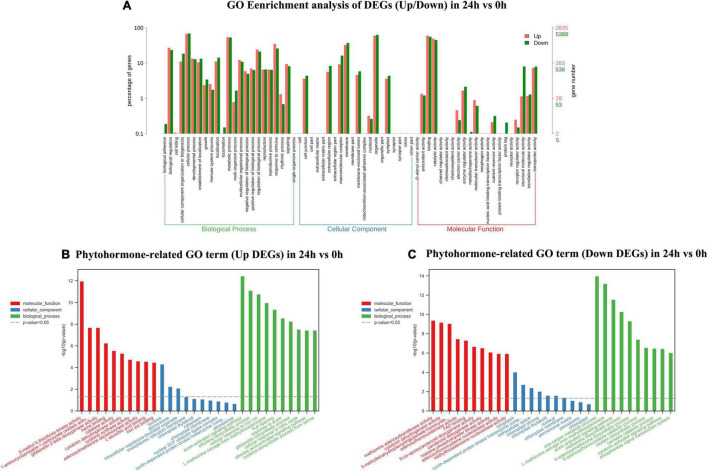
Gene ontology enrichment analysis of up/down DEGs **(A)**, phytohormone-related GO terms (up DEGs) **(B)**, and phytohormone-related GO terms (down DEGs) **(C)** in 24 vs. 0 h.

**FIGURE 5:**
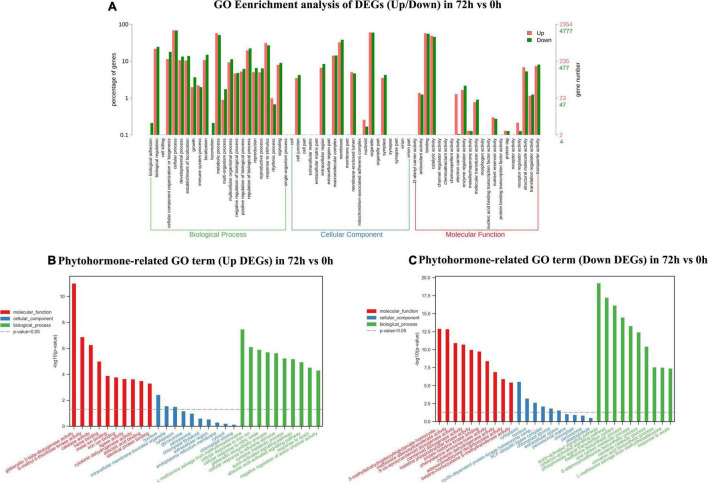
Gene ontology enrichment analysis of up/down DEGs **(A)**, phytohormone-related GO terms (up DEGs) **(B)**, and phytohormone-related GO terms (down DEGs) **(C)** in 72 vs. 0 h.

In 24 vs. 0-h and 72 vs. 0-h comparison, a total of 8,238 DEGs and 7,146 DEGs were, respectively, assigned into three main GO functional categories. The most abundant sub-categories of biological process, cellular component, and molecular function 24 vs. 0 h and 72 vs. 0 h were the same as that in 9 vs. 0 h (Figures 4A, 5A). Concerning the biological process, “response to stimulus” with 2,865 upregulated unigenes (34.78%) and 2,131 unigenes (25.87%) were displayed in the top 10 biological processes in 24 vs. 0 h (Figure 4A). In 72 vs. 0 h, the smaller number of the upregulated unigenes (2,228, 31.18%) involved in “response to stimulus” is displayed in Figure 5A when compared to that in 24 vs. 0 h. In plant hormone signal transduction and biosynthesis pathway, 253 EDGs in 24 vs. 0 h and 227 DEGs in 72 vs. 0 h were assigned into three main GO functional categories (Figures 4, 5). Generally, the number of downregulated DEGs related to the “auxin-activated signaling pathway” was increased to 22 unigenes with the elongation of waterlogging. Additionally, the upregulated DEGs in the “ethylene-activated signaling pathway” played an essential role in the biological process during the first day of waterlogging. The upregulated DEGs involved in the “abscisic acid-activated signaling pathway” remained high until the third day of waterlogging.

### The Gene Expression Associated With Phytohormones Biosynthesis Under Waterlogging Stress

**FIGURE 6:**
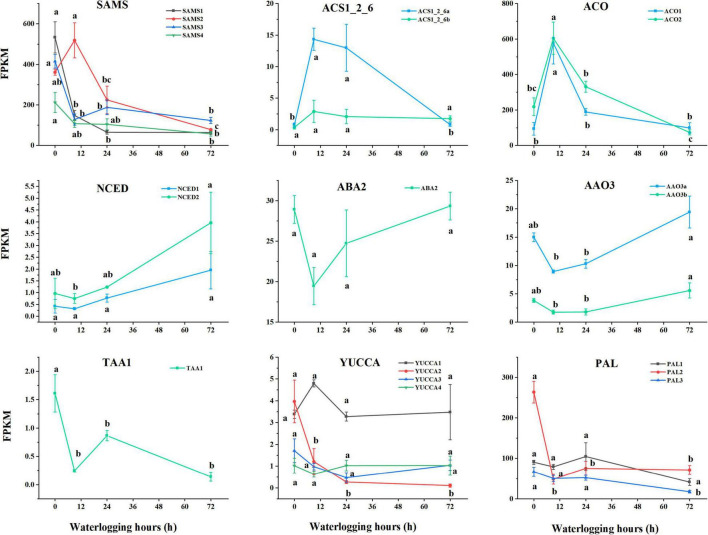
Dynamic patterns of related unigenes and transcription factors in phytohormones biosynthesis (ET, ABA, IAA, and SA). Data are means ± SD of three biological replicates. Abbreviations: ET: SAMS, *S*-adenosylmethionine synthetase [EC:2.5.1.6]; ACS1_2_6, 1-aminocyclopropane-1-carboxylate synthase 1/2/6 [EC:4.4.1.14]; ACO, aminocyclopropanecarboxylate oxidase [EC:1.14.17.4]; ABA: NCED, 9-cis-epoxycarotenoid dioxygenase [EC:1.13.11.51]; ABA2, xanthoxin dehydrogenase [EC:1.1.1.288]; AAO3, abscisic-aldehyde oxidase [EC:1.2.3.14]; IAA: TAA1, L-tryptophan—pyruvate aminotransferase [EC:2.6.1.99]; YUCCA, indole-3-pyruvate monooxygenase [EC:1.14.13.168]; SA: PAL, phenylalanine ammonia-lyase [EC:4.3.1.24].

Through KEGG pathway analysis, the unigenes related to the biosynthesis of ET, ABA, IAA, and SA were acquired in Figure 6. S-adenosylmethionine synthetase (SAMS, [EC:2.5.1.6]), 1-aminocyclopropane-1-carboxylate synthase (ACS, [EC:4.4.1.14]), and aminocyclopropanecarboxylate oxidase (ACO, [EC:1.14.17.4]) are the three core enzymes in the biosynthesis of ET in plants. We identified 47 unigenes encoding SAMS, 4 encoding ACS, and 7 unigenes encoding ACO. Except for several SAMS genes, the expression patterns of these unigenes mostly peaked in 9 h after waterlogging treatment. A number of three key enzymes including 9-cis-epoxycarotenoid dioxygenase (NCED, [EC:1.13.11.51]), xanthoxin dehydrogenase (ABA2, [EC:1.1.1.288]), and abscisic-aldehyde oxidase (AAO3, [EC:1.2.3.14]) are actively participated in ABA biosynthesis, which were encoded by 12 unigenes, 1 unigene and 2 unigenes, respectively. FPKM of NCED raised from 0.42–0.96 to 1.94–3.96 after experiencing 3-day waterlogging. The expression patterns of these unigenes showed a down–up trend. A number of two core enzymes, which are L-tryptophan—pyruvate aminotransferase (TAA1, [EC:2.6.1.99]) and indole-3-pyruvate monooxygenase (YUCCA, [EC:1.14.13.168]), play an essential role prior to IAA biosynthesis. FPKM of TAA1 dropped from 1.61 ± 0.33a to 0.14 ± 0.08b after 3-day waterlogging. *S. tonkinensis* seedlings carried out phenylalanine ammonia-lyase (PAL, [EC:4.3.1.24]) to generate SA in roots. We determined 6 unigenes encoding PAL with a downward trend after 3 days under waterlogging stress.

### The Comparison of Differentially Expressed Genes in Plant Hormone Signal Transduction Pathway Between Waterlogged Groups and the Control Group and Quantitative Real-Time PCR Analysis

After DEG acquisition, KEGG pathway significance analysis was conducted to classify these DEGs (Kanehisa et al., 2008). In the pathway of phytohormone signal transduction, a total of 48 positively regulated unigenes were identified and were targeted by 18 phytohormone-associated genes (ABF, 3 ARR-A, 2 BAK1, BIN2, 2 CTR1, EIN3, 3 ERF1, 4 ETR/ERS, 4 GH3, 10 [IAA], JAZ, PP2C, PR1, PYL, 5 SAUR, 5 SnRK2, 2 TCH4, and TGA) when comparing 9-h group and 0-h group. However, 33 unigenes were downregulated and were classified into 17 types of unigenes (ABF, 2 AHP, 2 ARR-B, 4 AUX1/LAX, BSK, DELLA, ETR/ERS, 5 [IAA], JAZ, 2 K14486/ARF, MYC2, 2 PP2C, 2 PR1, PYL, SAUR, 3 SnRK2, and 3 TGA) (Supplementary Figure 3 and Supplementary Table 1). A total of 11 DEGs and 14 DEGs in the auxin signaling transduction pathway were upregulated and downregulated, respectively (Supplementary Figure 6A). Compared to 0 h, 55 DEGs were upregulated and 25 genes were determined (2 ABF, 2 ARR-A, BAK1, BSK, BIN2, 2 CTR1, CYCD3, EIN3, 3 ERF1, 3 ETR/ERS, 3 GH3, GID1, 8 [IAA], JAR1_4_6, 2 JAZ, K14486/ARF, MYC2, 2 PP2C, 3 PR1, 2 PYL, 7 SAUR, 5 SnRK2, TCH4, TGA, and TIR1) in roots which had been waterlogged for 24 h, whereas 50 negatively regulated unigenes were sorted into 23 classifications associated with plant hormones (ABF, 4 AHP, 2 ARR-A, 2 AUX1/LAX, BRI1, BSK, 3 CYCD3, DELLA, EBF1_2, ETR/ERS, 2 GH3, 7 [IAA], 2 JAZ, 3 K14486/ARF, MYC2, NPR1, 4 PP2C, 2 PR1, 2 PYL, 3 SAUR, 3 SnRK2, TCH4, and TGA) (Supplementary Figure 4). Compared to the control group (0 h), the 72-h group owned 32 upregulated DEGs and 63 downregulated DEGs (Supplementary Figure 4). These DEGs were identified and sorted to 17 positively related classifications (2 ABF, BAK1, 2 BSK, EIN3, 2 ERF1, ETR/ERS, 3 GH3, GID1, [IAA], JAZ, K14486/ARF, 2 PP2C, 3 PR1, 2 PYL, 7 SAUR, SnRK2, and TGA) and 27 negatively related classifications (ABF, AHK2_3_4, 6 AHP, 4 ARR-A, 2 ARR-B, 4 AUX1/LAX, BAK1, BSK, COI-1, 3 CYCD3, DELLA, 2 EBF1_2, ERF1, ETR/ERS, 2 GH3, 9 [IAA], 2 JAZ, 4 K14486/ARF, MYC2, NPR1, 2 PP2C, 2 PR1, PYL, 6 SAUR, 2 SnRK2, TCH4, and TGA) (Supplementary Figure 5 and Supplementary Table 1). Generally, it was indicated that the number of significantly downregulated unigenes was increased with the time extension of waterlogging, especially in the auxin signal transduction pathway (Supplementary Figure 6 and Supplementary Table 1).

Based on the results of our transcriptomic analysis, eight genes known to be involved in phytohormones signal transduction were selected to confirm their mRNA expression levels by qRT-PCR. The results of qRT-PCR are displayed in Supplementary Figure 7, which were consistent with their variation trends in transcriptional profiles.

## Discussion

### Ethylene Biosynthetic and Ethylene-Responsive Genes

Ethylene is an essential phytohormone, which performs as a chemical messenger in many physiological processes throughout a plant life cycle (Schaller and Voesenek, 2015). It also gets involved in a wide range of combating responses to abiotic and biotic stresses (Müller and Munné-Bosch, 2015). Oxygen deprivation around roots caused by waterlogging induces ET accumulation in most plant species (Voesenek et al., 2003; Jackson, 2008; Alpuerto et al., 2016). Under waterlogging conditions, ET improves plant adaption to flooding by triggering the formation of adventitious roots in tomatoes (Vidoz et al., 2016), playing an important role in the ROS-signaling pathway and antioxidant system (Khan et al., 2020). Coudert et al. (2010) demonstrated that ET facilitated the formation of adventitious roots in rice. In the process of ET biosynthesis, methionine is catalyzed by SAMS to synthesize s-adenosylmethionine (SAM). Subsequently, SAM is carboxylated by ACS into ACC, followed by the oxidization of ACC to form ET under ACO regulation (Sauter et al., 2013; Chen et al., 2016). As indicated in Figure 6, the expression level of ACO (E1.14.17.4) peaked at 9 h after waterlogging, followed by a downward trend with the limited O_2_. It was suspected that the ACC oxidation process was enforced at the early stage to generate ET, whereas ACC started to massively accumulate under hypoxia conditions with the low expression of ACO (Figure 1A).

Commonly, the ET receptor (ETR/ERS) can stimulate the kinase activity of constitutive triple-response 1 (CTR1) without ET. The activated CTR1 will repress the activity of ET insensitive 3 (EIN3) transcription factors (Guo and Ecker, 2003; Alonso and Stepanova, 2004; Chen et al., 2005). In our study, both CTR1 and ETR/ERS kept the same trend of obvious upregulation throughout waterlogging process (Supplementary Table 1). Previous studies identified two F box proteins which were called EBF1 and EBF2 in *Arabidopsis*, respectively. EBF1 and EBF2 mediate ubiquitination or proteasome pathway to negatively regulate ET signal transduction. EBF1_2 also called EIN3-binding F-box protein leads to degradation of EIN3 when EBF1_2 is overexpressed, consequently causing plants insensitive to ET (Guo and Ecker, 2003; Potuschak et al., 2003), which is the opposite to the results in Supplementary Table 1. EIN3 is an important positive regulator of the downstream ET pathway. The stabilization of EIN3 also refers to the ability to rapidly sense hypoxia signaling and activate the ET pathway in the early stage (Chao et al., 1997; Xie et al., 2015). This discovery was consistent with the results that DEGs related to the “ethylene-activated signaling pathway” were induced under waterlogging during the first day in Figures 3, 4. Genetic analysis proved that ET response factor 1 (ERF1) belongs to the AP2/ERF superfamily and participates downstream of EIN3 and other ET pathway components. ERF1, a positive ET-responsive gene, also can be regulated by EIN3 (Chao et al., 1997; Ma et al., 2014). In our study, EIN3 was positively regulated since the downregulated EBF1_2 cannot suppress the expression of EIN3, further activating the role of ERF1 in the downstream “ethylene-activated signaling pathway” (Guo and Ecker, 2003; Alonso and Stepanova, 2004; Chen et al., 2005). In general, the upregulated EIN3 indicated the ability to rapidly sense hypoxia signaling and activate the ET pathway in *S. tonkinensis* seedlings at the early stage of waterlogging (Chao et al., 1997; Xie et al., 2015).

### Abscisic Acid Biosynthetic and Abscisic Acid-Responsive Genes

Abscisic acid is a critical plant hormone for plant growth, which can accumulate in plants in response to adversity by improving turgor pressure (Vishwakarma et al., 2017). However, the results revealed that ABA accumulation in the roots of *S. tonkinensis* seedlings has been inhibited (Figure 1B). During ABA biosynthetic procedure, 9-cis-violaxanthin is prepared to produce xanthoxin under the catalysis of NCED. Then, ABA2 induces the dehydrogenation of xanthoxin to form abscisic aldehyde, which is eventually oxidized to synthesize ABA under AAO3 catalysis (Nambara and Poll, 2005). The gene expression of ABA biosynthesis-related unigenes took on a down–up trend (Figure 6), which was consistent with the dynamic pattern of ABA content in Figure 1B.

Abscisic acid can be perceived by its receptor proteins from the pyrabactin resistance 1-like (PYL) family, further sequestrating type 2C protein phosphatases (PP2C) while activating sucrose non-fermenting 1-related protein 2 (SnRK2). Subsequently, ABA signaling involves phosphorylation of the downstream ABRE-binding factor (ABF), which recognizes and regulates the expression of ABA-responsive genes, thereby enhancing the resistance of plants at expense of growth (Fujii et al., 2009; Ma et al., 2009; Zhao Y. et al., 2018; Belda-Palazón et al., 2020; Cui et al., 2020). As shown in Supplementary Table 1, PYL was negatively regulated, leading to weak suppression of PP2C and negatively regulated SnRK2 in roots of *S. tonkinensis* seedlings. It was inferred that upregulated ABF could recognize and regulate ABA in roots of *S. tonkinensis* seedlings under flooding stress (Mustilli et al., 2002; Yoshida et al., 2006; Fujii et al., 2007; Umezawa et al., 2009; Vlad et al., 2009).

### Indole-3-Acetic Acid Biosynthetic and Auxin-Responsive Genes

Auxins (e.g., IAA) are known to be the major phytohormones involved in the process of the formation of adventitious roots, which is a vital adaptive trait under waterlogging stress (Vidoz et al., 2010; Zhang et al., 2015). Morris (1993) demonstrated that IAA played an important role in regulating plant growth and development to cope with hypoxia. The variation of IAA in roots of *S. tonkinensis* had an upward trend first and a downward trend subsequently on the third day (Figure 1C). The IAA biosynthesis process is a part of tryptophan metabolism. TAA1 uses tryptophan to form indole pyruvic acid (Stepanova et al., 2008). Mashiguchi et al. (2011) reported that the YUC gene family catalyzes the direct conversion of indole-3-pyruvic acid monohydrate to IAA. Won et al. (2011) discovered that TAA1 and YUC genes play a synergistic role in auxin biosynthesis. The expression level of unigenes related to IAA biosynthesis presented the fluctuation. Generally, FPKM of TAA1 and YUCCA at 72 h after waterlogging was decreased when compared to the control group, indicating the declined content of IAA in the 72-h group (Figure 1C).

Previous studies indicated that auxin-responsive genes (Aux/IAA, GH3, and SAUR) and auxin response factor genes (ARFs) participated in the process of the “auxin-activated signaling pathway” to strengthen plant resistance to abiotic stress (Hagen and Guilfoyle, 2002; Xie et al., 2020). ARFs are combined with Aux/IAA transcriptional repressors when auxin concentration is low, thereby inhibiting auxin-responsive genes (GH3 and SAUR). With the increase of auxin, transport inhibitor response 1 (TIR1) starts to recruit Aux/IAA, contributing to the ubiquitination and degradation of Aux/IAA (Dharmasiri et al., 2005; Mockaitis and Estelle, 2008; Maraschin et al., 2009). Subsequently, ARFs are released with proteolysis, activating auxin transcription (Leyser, 2018). Li et al. (2020) also reported that SAUR72, ARF6, and [IAA] genes were significantly upregulated whereas the GH3 gene was significantly downregulated in waterlogged plants. Nonetheless, Zhang et al. (2016) discovered that several auxin-related genes were negatively regulated in leaves of waterlogged cotton. Further, Kramer and Ackelsberg (2015) found that ARF17 was positively correlated with GH3 in the leaves and roots of *Populus deltoides* “DHY,” which was opposite to the consequences in Supplementary Table 1. In the first two groups, SAUR, GH3, and [IAA] genes were upregulated whereas K14486/ARF and Auxin influx carrier (AUX1/LAX) were downregulated. AUX1/LAX plays an essential role in the polar auxin transport (PAT), which is responsible for transporting auxin from root tips to the elongation zone (Péret et al., 2012).

### Salicylic Acid Biosynthetic and Salicylic Acid-Responsive Genes

Salicylic acid, a chemical messenger, can be synthesized in abundance in plants to improve plant tolerance under abiotic and biotic stresses. It is a β-hydroxy phenolic acid, which induces growth, development, and biochemical traits of plants at even minuscule concentrations (Arif et al., 2020). Miura and Tada (2014) expounded that SA physiologically got involved in plants by inducing stomatal closure and regulating plant morphology. The shikimic acid pathway is one of the synthetic routes for SA. Phenylalanine originated from shikimic acid and is catalyzed by PAL to synthesize trans-cinnamic acid (trans-CA) (Mishra and Baek, 2021). Subsequently, trans-CA is converted to o-coumaric acid (o-CA), and o-CA is directly converted to SA (León et al., 1995). The expression trend of PAL was identical to the dynamic pattern of SA content (Figure 1D).

In this study, three SA-responsive genes (PR1, TGA, and NPR1) participated in SA signaling pathway. Johnson et al. (2003) revealed that transcription factor TGA (TGA) stimulated the pathogenesis-related protein 1 (PR1) promoter *in vivo* in the SA and regulatory protein NPR1 (NPR1)-dependent manner. Besides, the gene expression of PR is regulated by NPR1 with transcription factors such as TGA (Zhou et al., 2000; Kim and Delaney, 2002). Ali et al. (2018) claimed that PR genes get significantly altered under adversities. This was consistent with our results that PR1 was significantly altered between waterlogging samples and the control group (0 h) (Supplementary Table 1).

### Crosstalk Between Phytohormones Under Waterlogging

The plant perception of abiotic stress triggers the crosstalk between phytohormones in response to adversities. This crosstalk forms a signaling network (Harrison, 2012). In the study, ACC showed an upward trend whereas ABA, IAA, and SA took on a downward trend from 0 to 72 h under waterlogging stress. Yamauchi et al. (2020) disclosed that AUX/IAA-mediated auxin signaling possibly plays a role in ET-dependent aerenchyma formation in rice roots under hypoxia conditions. As an alternative to the accumulation of ABA, ET may act as a root-sourced messenger transported as ACC in water stress circumstances (Jackson, 1997). Sharp (2002) proposed that endogenous ABA inhibited ET generation under anaerobic conditions. The interaction between ABA and ET contributes to the maintenance of shoot and root growth (Sharp, 2002). Yin et al. (2015) pointed out that ET and ABA seem to act synergistically or antagonistically to control plant growth and development. Benschop et al. (2005) and Saika et al. (2007) expounded that accumulated ET was not only an obstacle to NCED expression but also caused a breakdown of ABA into phaseic acid, thereby decreasing ABA. Table 5 indicates that waterlogging had an opposite effect on the content of ET precursor (ACC) and ABA (r = −0.973). Previous research found ABA accumulation to foster stomatal closure (Savchenko et al., 2014). On the contrary, Chini et al. (2007) and Thines et al. (2007) explained that auxins regulated stomatal opening positively. ABA can cooperate with auxin to facilitate plant growth, owing to that ABA treatment improves the gene expression of ARF2 but decreases IAA7 to activate the auxin signaling pathway (Song et al., 2009; Wang et al., 2011). In this study, K14486/ARF was downregulated, but [IAA] was upregulated with the reduction of ABA content (Figure 1B and Supplementary Table 1). Combined with the decreased IAA content, it was concluded that waterlogging had a similar impact on the variation of ABA and IAA content (*r* = 0.982) in *S. tonkinensis* (Table 5).

**TABLE 5:** The correlations between 1-aminocyclopropane-1-carboxylic acid (ACC), abscisic acid (ABA), indole-3-acetic acid (IAA), and salicylic acid (SA) in 0- and 72-h groups.

Phytohormones	ACC	ABA	IAA	SA
ACC	1	−0.973**	−0.940**	−0.973**
ABA	−0.973**	1	0.982**	0.950**
IAA	−0.940**	0.982**	1	0.957**
SA	−0.973**	0.950**	0.957**	1

**Indicate significant differences (p < 0.01) among the phytohormones.

**FIGURE 7:**
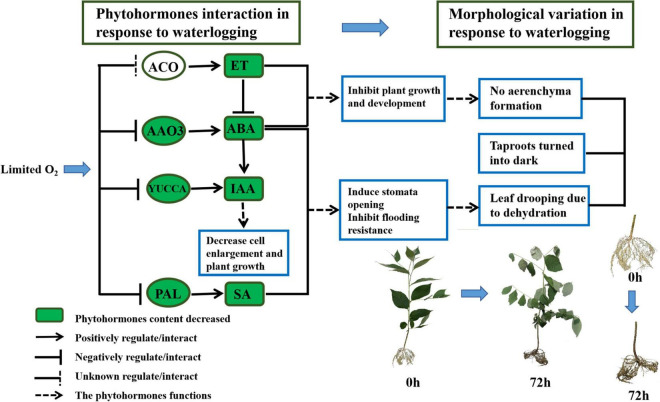
Schematic diagram of phytohormones crosstalk (72 vs. 0 h) and morphological variation (72 vs. 0 h) in response to waterlogging in *Styrax tonkinensis* seedlings. Different lowercase letters within each graph indicate significant differences (*p* < 0.05) among the samplings.

Overall, the upregulated EIN3 and downregulated EBF1_2 assisted in ethylene signaling at an early stage and kept *S. tonkinensis* seedlings sensitive to ET. Nonetheless, ET biosynthesis was inhibited by the low expression level of ACO on the third day of waterlogging. In addition, the “ET activated signaling pathway” failed to form adventitious roots and aerenchyma in *S. tonkinensis* seedlings (Figure 7). The “auxin-activated signaling pathway” was enriched in both the upregulated and downregulated genes in 9 vs. 0 h and 24 vs. 0 h, whereas it was only enriched in the downregulated genes in 72 vs. 0 h. The falling expression level of PAL resulted in the low accumulation of SA content at 72 h (Figure 7). Moreover, the decreased transcription of PYL weakened the ABA signal transduction pathway in the roots of *S. tonkinensis* seedlings under waterlogging stress.

One limitation in our study is the large biological variability since these seedlings originated from seeds. In the future, spraying exogenous ABA and SA on the seedlings from cutting propagation will be conducted to reinforce the suspected conclusion. Also, further exploration of the interaction of these genes plays an essential role in the understanding of the crosstalk between phytohormones and theoretically supports the molecular mechanism of *S. tonkinensis* seedlings’ resistance to waterlogging.

## Conclusion

To understand the phytohormone regulatory mechanisms in *S. tonkinensis* seedling roots under flooding stress, we determined the variation of ACC and three phytohormones content (ABA, IAA, and SA) in roots of *S. tonkinensis* seedlings in response to waterlogging stress after 0, 9, 24 and 72 h, respectively. It was found that ACC showed an upward trend, but other plant hormones showed a downward trend from 0 to 72 h under waterlogging stress. We also discovered several DEGs involved in phytohormone synthesis pathway and plant hormone signaling pathway. It was concluded that the decreased transcription of PYL weakened the ABA signal transduction pathway in the roots of *S. tonkinensis* seedlings under waterlogging stress. Moreover, decreased SA content caused by the low-expressed PAL reduced the resistance of *S. tonkinensis* seedlings under waterlogging stress.

## Data Availability Statement

The datasets presented in this study can be found in online repositories. The names of the repository/repositories and accession number(s) can be found below: National Center for Biotechnology Information (NCBI) BioProject database under accession number PRJNA817050.

## Author Contributions

HC contributed to conceptualization, software, formal analysis, investigation, writing original draft, and editing the manuscript. QW contributed to data curation, reviewing, and editing the manuscript. MN contributed to conceptualization, resources, and investigation. CC contributed to the investigation and editing the manuscript. CH contributed to the investigation. FY contributed to conceptualization, funding acquisition, supervision, reviewing, and editing the manuscript. All authors contributed to the article and approved the submitted version.

## Conflict of Interest

The authors declare that the research was conducted in the absence of any commercial or financial relationships that could be construed as a potential conflict of interest.

## Publisher’s Note

All claims expressed in this article are solely those of the authors and do not necessarily represent those of their affiliated organizations, or those of the publisher, the editors and the reviewers. Any product that may be evaluated in this article, or claim that may be made by its manufacturer, is not guaranteed or endorsed by the publisher.

## Supplementary Material

Supplementary Figure 1 **(A)** The detailed design of waterlogging experiment. **(B)** The primary root part displayed in red frame was isolated from the whole root for the following experiments.

Supplementary Figure 2 Quality control of different samples from different biological replicates in the pathway of plant hormone signal transduction. **(A)** The box-whisker plot of each sample in the pathway of plant hormone signal transduction of *S. tonkinensis* seedlings under waterlogging stress. The box-whisker plot for each region has five statistics (maximum, third quartile, median, first quartile, and minimum). **(B)** Principal component analysis (PCA) of root samples of *S. tonkinensis* seedlings collected at 0, 9, 24, and 72 h.

Supplementary Figure 3 Hierarchical cluster analysis (HCA) of unigenes comparison between 9 and 0 h in the pathway of plant hormone signal transduction of *S. tonkinensis* seedlings under waterlogging stress. Red blocks represent high expression of unigenes, and blue blocks represent a low expression of unigenes. These unigenes in the figure above need to meet the threshold that *p* < 0.05 and | log2FC| > 1.

Supplementary Figure 4 Hierarchical cluster analysis of unigenes comparison between 24 and 0 h in the pathway of plant hormone signal transduction of *S. tonkinensis* seedlings under waterlogging stress. Red blocks represent a high expression of unigenes, and blue blocks represent a low expression of unigenes. These unigenes in the figure above need to meet the threshold that *p* < 0.05 and | log2FC| > 1.

Supplementary Figure 5 Hierarchical cluster analysis of unigenes comparison between 72 and 0 h in the pathway of plant hormone signal transduction of *S. tonkinensis* seedlings under waterlogging stress. Red blocks represent a high expression of unigenes, and blue blocks represent a low expression of unigenes. These unigenes in the figure above need to meet the threshold that *p* < 0.05 and | log2FC| > 1.

Supplementary Figure 6 The up- or downregulation of DEGs in plant hormone signal transduction pathway sorted by gene functions.

Supplementary Figure 7 The relative expression from qRT-PCR of IAA **(A)**, PYL **(B)**, EBF1_2 **(C)**, EIN3 **(D)**, MYC2 **(E)**, NPR1 **(F)**, AHP **(G)**, and AUX1/LAX **(H)** in roots of *S. tonkinensis* seedlings under waterlogging stress after 0, 9, 24, and 72 h, respectively. Different lowercase letters within each graph indicate significant differences (*p* < 0.05) among the samplings, whereas different capital letters within each graph indicate significant differences (*p* < 0.01) among the samplings.
