# Supplementary figures and images for: Transcriptome Analysis of Endogenous Hormone Response Mechanism in Roots of Styrax tonkinensis Under Waterlogging
Source: Front Plant Sci. 2022 Jun 6;13:896850. doi: 10.3389/fpls.2022.896850 (PMC9208659; doi:10.3389/fpls.2022.896850)

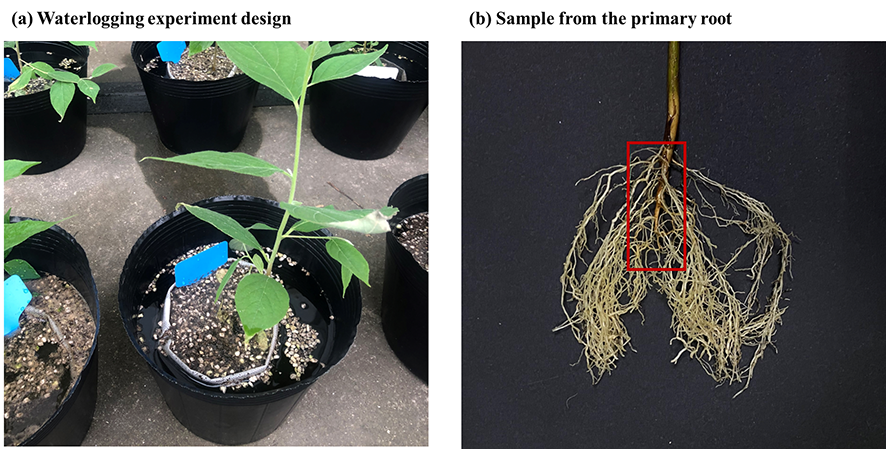

Supplement: Supplementary Figure 1 — (A) The detailed design of waterlogging experiment. (B) The primary root part displayed in red frame was isolated from the whole root for the following experiments. [file Image_1.TIF]

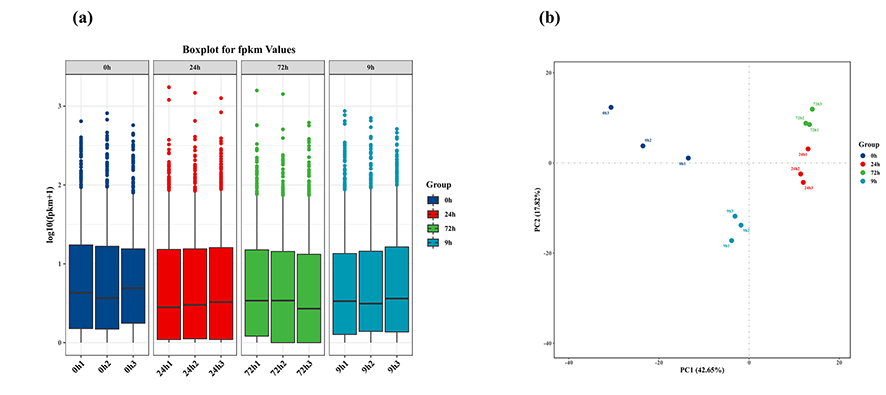

Supplement: Supplementary Figure 2 — Quality control of different samples from different biological replicates in the pathway of plant hormone signal transduction. (A) The box-whisker plot of each sample in the pathway of plant hormone signal transduction of S. tonkinensis seedlings under waterlogging stress. The box-whisker plot for each region has five statistics (maximum, third quartile, median, first quartile, and minimum). (B) Principal component analysis (PCA) of root samples of S. tonkinensis seedlings collected at 0, 9, 24, and 72 h. [file Image_2.TIF]

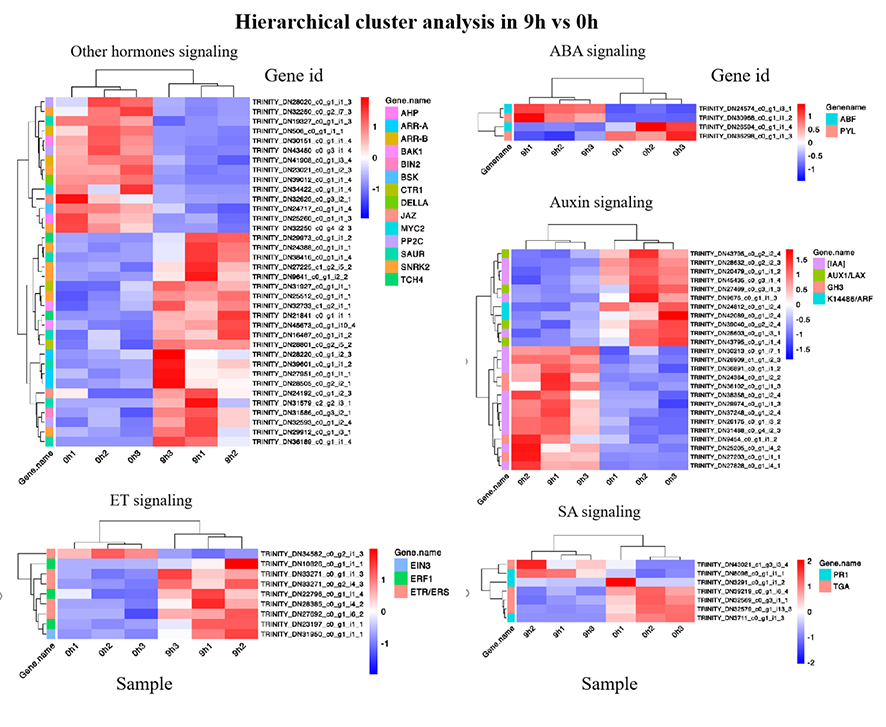

Supplement: Supplementary Figure 3 — Hierarchical cluster analysis (HCA) of unigenes comparison between 9 and 0 h in the pathway of plant hormone signal transduction of S. tonkinensis seedlings under waterlogging stress. Red blocks represent high expression of unigenes, and blue blocks represent a low expression of unigenes. These unigenes in the figure above need to meet the threshold that p < 0.05 and | log2FC| > 1. [file Image_3.TIF]

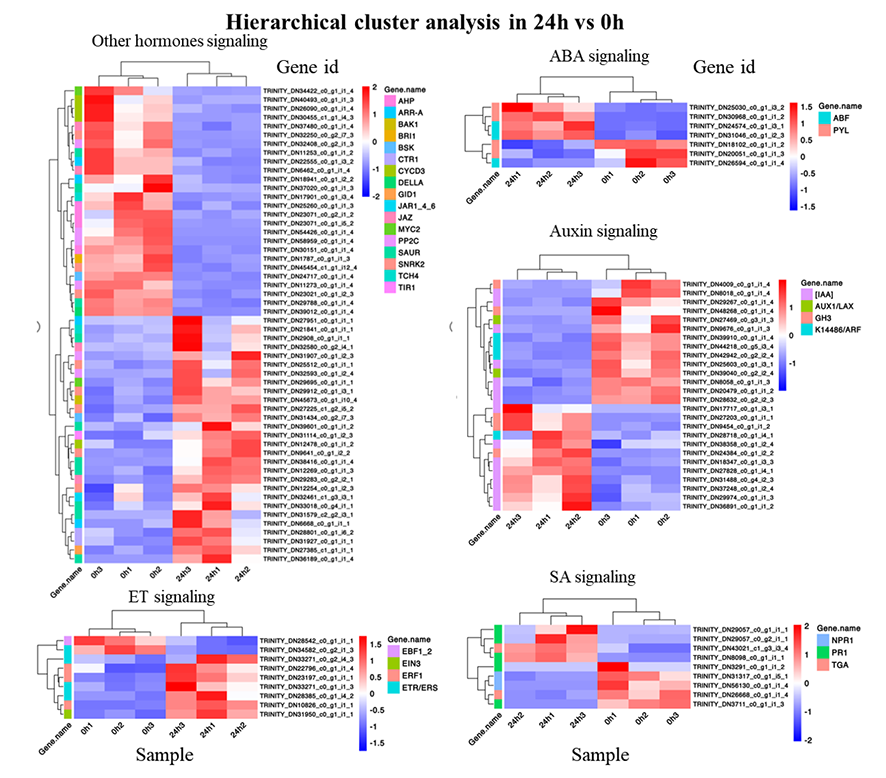

Supplement: Supplementary Figure 4 — Hierarchical cluster analysis of unigenes comparison between 24 and 0 h in the pathway of plant hormone signal transduction of S. tonkinensis seedlings under waterlogging stress. Red blocks represent a high expression of unigenes, and blue blocks represent a low expression of unigenes. These unigenes in the figure above need to meet the threshold that p < 0.05 and | log2FC| > 1. [file Image_4.TIF]

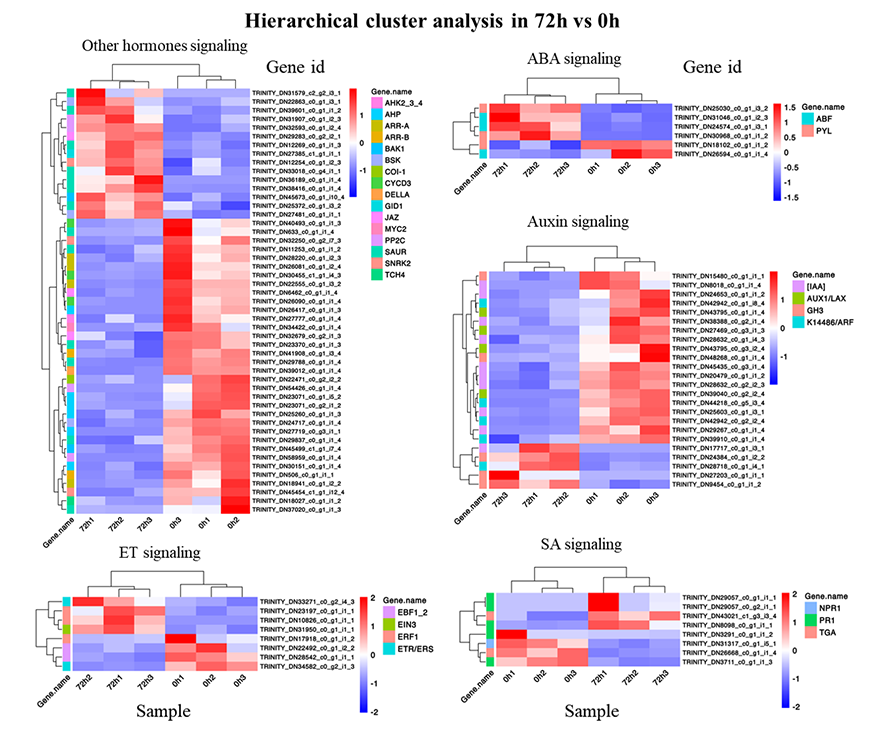

Supplement: Supplementary Figure 5 — Hierarchical cluster analysis of unigenes comparison between 72 and 0 h in the pathway of plant hormone signal transduction of S. tonkinensis seedlings under waterlogging stress. Red blocks represent a high expression of unigenes, and blue blocks represent a low expression of unigenes. These unigenes in the figure above need to meet the threshold that p < 0.05 and | log2FC| > 1. [file Image_5.TIF]
